# Systematic review and modelling of *Toxoplasma gondii* seroprevalence in humans, Europe, 2000 to 2021

**DOI:** 10.2807/1560-7917.ES.2025.30.34.2500069

**Published:** 2025-08-28

**Authors:** Ingrid HM Friesema, Helga Waap, Arno Swart, Adriana Györke, Delphine Le Roux, Francisco MD Evangelista, Furio Spano, Gereon Schares, Gunita Deksne, Maria João Gargaté, Rafael Calero-Bernal, Pikka Jokelainen, Frank Seeber, Jacek Sroka, Anna Lundén, Oda van den Berg, Solveig Jore, Henk J Wisselink, Filip Dámek, Lasse S Vestergaard, Marieke Opsteegh

**Affiliations:** 1 Centre for Infectious Disease Control, National Institute for Public Health and the Environment, Bilthoven, The Netherlands; 2 Department of Parasitology and Parasitic Diseases, Faculty of Veterinary Medicine, University of Agricultural Sciences and Veterinary Medicine Cluj-Napoca, Cluj-Napoca, Romania; 3 Anses, INRAE, Ecole Nationale Vétérinaire d’Alfort, Laboratoire de Santé Animale, BIPAR, Maisons-Alfort, France; 4 Department of Comparative Biomedical Sciences, School of Veterinary Medicine, Faculty of Health and Medical Sciences, University of Surrey, Guildford, United Kingdom; 5 Unit of Foodborne and Neglected Parasitic Diseases, Department of Infectious Diseases, Istituto Superiore di Sanità, Rome, Italy; 6 Institute of Epidemiology, Friedrich-Loeffler-Institut, Federal Research Institute for Animal Health, Greifswald, Germany; 7 Institute of Food Safety, Animal Health and Environment “BIOR”, Riga, Latvia; Faculty of Medicine and Life Sciences, University of Latvia, Riga, Latvia; 8 Department of Infectious Diseases, National Institute of Health Dr. Ricardo Jorge (INSA), Lisbon, Portugal; 9 SALUVET, Animal Health Department, Faculty of Veterinary Sciences, Complutense University of Madrid, Madrid, Spain; 10 Infectious Disease Preparedness and One Health, Statens Serum Institut, Copenhagen, Denmark; 11 FG 16: Mycotic and parasitic agents and mycobacteria, Robert Koch-Institute, Berlin, Germany; 12 Department of Parasitology and Invasive Diseases, Bee Diseases and Aquatic Animal Diseases, National Veterinary Research Institute, Pulawy, Poland; 13 Department of Microbiology, Swedish Veterinary Agency, Uppsala, Sweden; 14 Zoonotic and Waterborne infections, Norwegian Institute for Public Health (NIPH), Oslo, Norway; 15 Wageningen Bioveterinary Research, Wageningen University and Research, Lelystad, the Netherlands; 16 Department of Infectious Disease Epidemiology and Prevention, and Department of Bacteriology, Parasitology and Mycology, Statens Serum Institut, Copenhagen, Denmark; 17 Egas Moniz Center for Interdisciplinary Research (CiiEM), Egas Moniz School of Health & Science, Caparica, Portugal; 18 Laboratório de Parasitologia, Instituto Nacional de Investigação Agrária e Veterinária, Oeiras, Portugal

**Keywords:** Europe, Human, Toxoplasma gondii, Seroprevalence, Systematic review

## Abstract

**BACKGROUND:**

*Toxoplasma gondii* is a zoonotic protozoan capable of infecting warm-blooded animal species and humans. Although toxoplasmosis presents mostly as mild or asymptomatic infection in immunocompetent individuals, in unborn children and people with weakened immune systems, the disease can be severe with ocular, neurological or multi-systemic manifestations and even death.

**AIM:**

We aimed to collate and analyse data on *T. gondii* seroprevalence in humans to model and compare age-dependent prevalence in geographic regions in Europe.

**METHODS:**

A systematic review identified 1,822 scientific publications, from which seroprevalence data were extracted from 69 studies. Data were analysed using a Bayesian hierarchical model.

**RESULTS:**

The modelling of the seroprevalence indicated the highest incidence rates in eastern (50%) and western (48%) Europe, with the lowest estimates in northern Europe (18%) and the United Kingdom (UK) (18%). Eastern and western Europe were regions where *T. gondii* infections occurred earliest in life, with half of the population expected to be seropositive by the age of 44 and 47 years, respectively. In contrast, in northern Europe and the UK the modelled median time to infection exceeded 170 years.

**CONCLUSION:**

Results of the study provide a robust baseline for future epidemiological research on human *T. gondii* infections in Europe and may be useful to validate subsequent research, such as risk assessment studies.

## Introduction

*Toxoplasma gondii*, a protozoan parasite with a worldwide distribution, is capable of infecting humans and potentially all warm-blooded vertebrates [1]. Felids serve as the definitive hosts for *T. gondii* [2]. When ingested, the parasite replicates in the felid’s intestine, followed by shedding of the oocysts via faeces into the environment. The oocysts can sporulate and survive for long periods in the environment [3]. Ingestion of sporulated *T. gondii* oocysts present in contaminated water, soil or fresh produce can lead to formation of tissue cysts in all susceptible hosts, including humans [4]. The bradyzoites in these tissue cysts are infective, allowing transmission through the consumption of undercooked or raw meat from infected hosts [5,6]. Humans can become infected both via the environmental route and via consumption of undercooked or raw meat of infected animals. Another route is transplacental transmission to a fetus, causing congenital infection potentially resulting in abortion or stillbirth [7]. Moreover, *T. gondii* can be transmitted via blood transfusions or organ transplants [5,8].

Acquired *T. gondii* infections in humans are generally asymptomatic or cause non-specific and self-limiting symptoms, but they can also present as ocular toxoplasmosis. Severe acute toxoplasmosis, although rare, may manifest as myocarditis, polymyositis, pneumonitis, retinitis, hepatitis or encephalitis, and mainly occurs in people with severely weakened immune systems [5]. Prevalence of *T. gondii* infection is influenced by factors such as climate, cultural habits such as consumption of raw meat, hygiene practices and socioeconomic conditions [9].

Serological testing for *T. gondii* infection in humans is performed for several reasons. In some countries, screening is conducted during pregnancy to monitor seroconversion and guide treatment. Testing also helps to assess population seroprevalence and aids in diagnosing clinical toxoplasmosis. Both, infection with *T. gondii* and the presence of detectable antibodies, are assumed to persist lifelong, with the prevalence of anti-*T. gondii* IgG in humans increasing with age [9].

The aim of this work was to collate available published data on human *T. gondii* seroprevalence to model and compare the age-dependent prevalence of the infection in Europe.

## Methods

### Literature screening and study selection

A structured literature search was carried out according to the PRISMA guidelines [10], using Emtree terms within the Embase literature database. The search string can be found in Supplementary Table S1. The search terms were chosen to cover human seroprevalence and risk factors of infections with *T. gondii* in Europe. For the study area, 41 countries in Europe were considered, including the 27 European Union (EU) countries. The list of the countries included is presented in Supplementary Table S2. The publication period of interest was set from January 2000 to May 2021. The search strategy had no limitations on publication language. The search was conducted in May 2021.

A group of 17 scientists with expertise in *T. gondii* and toxoplasmosis from 12 countries across Europe assessed the eligibility of the publications identified. The screening of the publications was performed within Cadima [11], an open-access online software tool for conducting systematic reviews. The systematic review was done in two stages based on a set of predefined criteria: an article was eligible if (i) it reported a study based on original data; (ii) the study was on human seroprevalence and/or risk factors for *T. gondii* infection in the included European countries, with at least part of the data collected from the year 2000 onwards; and (iii) had been published in a peer-reviewed journal. Reviews, meta-analyses and other articles not reporting original data were excluded, as were studies investigating the prevalence of *T. gondii* in particular risk groups and studies investigating *T*. *gondii* infection as a risk factor for another condition. First, the title and abstract were screened by two randomly chosen scientists from the group, after which consensus on inclusion had to be reached in case of initial disagreement. All inconsistencies were solved between the two scientists without the need of a third person. Second, full text of the remaining publication was screened and again consensus on inclusion was reached. The same criteria were used in both screening steps.

The next step involved extracting relevant data from the selected articles. Per article, data were extracted by one scientist and then checked by another. Thirteen scientists from the previous group were involved in this process, and four additional scientists helped extracting data from articles in different languages. The data were gathered in a template file created in Microsoft Excel. For each study, data on study design, period, population, serological tests used, and results were registered. Extracted data on seroprevalence were harmonised and categorised for modelling. For this, countries were assigned to one of five European regions (western, northern, eastern, southeastern, southwestern), as described previously [12,13].

### Data analysis

Some publications presented more than one set of results on seroprevalence for the same population (e.g. results for the total study population, as well as for various subcategories based on, for example, age, sex and/or region), resulting in several rows of data for those populations. In those cases, the subpopulations were weighted by their probability to ensure that they contributed proportionally to the actual number of participants in the study. The meta-analysis was done using the data with specification on age, as reported in the publications. Since the exact ages of individual participants at the time of sampling were not provided, we defined an uncertainty distribution based on the estimates of the minimum, maximum and most probable age at sampling. If a median or mean age was given per age range, this was used as most probable age; otherwise, the median age was calculated from the minimum and maximum age of the age range. Data from studies that did not specify age of the participants or only reported data for all ages combined were excluded from data analysis.

A Bayesian hierarchical model built for estimating the age-dependent seroprevalence of *T. gondii* in animal species [13] was adapted to the human data collected in the present review. This model consists of an age-dependent Susceptible-Infected-Susceptible (SIS) framework, where individuals move from susceptible (i.e. seronegative) to infected (i.e. seropositive), with the possibility of reversion to susceptible (i.e. loss of detectable antibody response). Individuals were considered born susceptible, and hence able to move into the infected compartment based on a constant force of infection ( λ , incidence rate, i.e. the rate at which individuals acquire infection measured in new infections per year) and with the reversion to seronegative at rate γ.

The Bayesian hierarchical model was built to be able to estimate the parameters through partial pooling, granting the possibility to overcome data gaps. Variables used in the model are shown in Table 1.

**Table 1:** Data used in the Bayesian hierarchical model of *Toxoplasma gondii* seroprevalence in humans, Europe, 2000–2021

Variable^a^	Values
region[i]	Eastern, Northern, Southeastern, Southwestern, Western Europe
pop[i]	A unique identifier for a population
ntot[i]	Total number of participants tested
npos[i]	Total number of participants test positive
agemin[i]	Lower bound of the age range
agemax[i]	Upper bound of the age range
agemean[i]	The most probable age at sampling

^a^ Variables as included in the model with corresponding values and the index [i] corresponding to the i-th data point.

In the process of model fitting by means of Bayesian inference, the age distribution for each population was updated, i.e. a posterior age distribution was obtained. Differences between regions were considered using a hierarchical model and modelled as linear contributions to the logarithmic baseline force of infection (λ). No regional differences were considered for the reversion rate (γ). Model fitting was performed using Stan (https://mc-stan.org) (interfaced with R version 4.1.3 (https://www.r-project.org/). Trace plots of the Markov chains were visually assessed to confirm the convergence of the model.

## Results

### Data collection

A total of 1,822 publications were identified, of which 12 were removed as duplicates. After screening titles and abstracts, 367 articles were selected for full-text screening, of which 142 articles met the inclusion criteria. During data extraction, a further 67 articles were excluded because of missing or incomplete information. Of the remaining 75 publications, 69 provided seroprevalence data [14-83] and 22 contained data on risk factors. A PRISMA flow diagram is presented in Supplementary Figure S1, and a list of the 22 references is also included in the Supplementary Material. The further analyses focused solely on seroprevalence. Relevant seroprevalence data could be recovered from 25 of the 41 countries considered in the search strategy.

### Regional seroprevalence of *Toxoplasma gondii* in Europe

**Figure 1:**
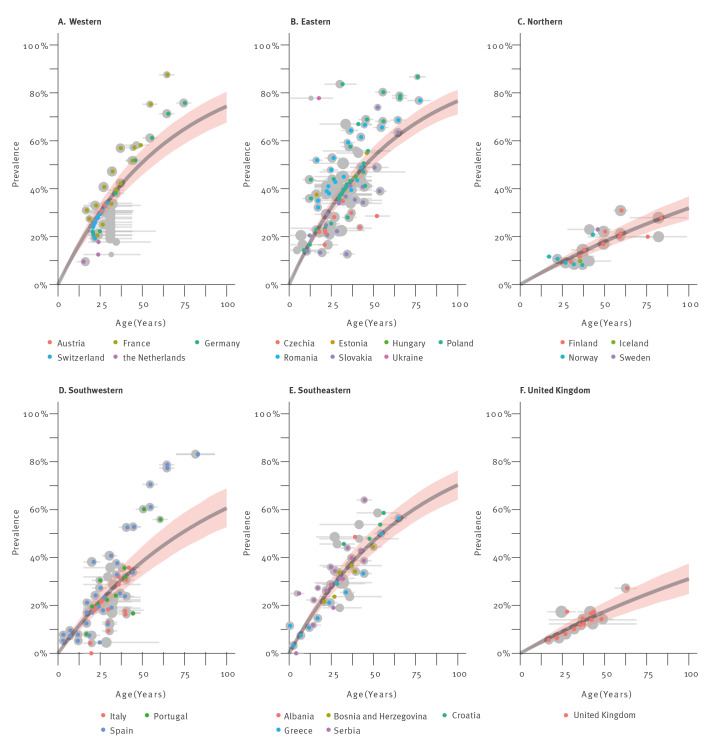
Susceptible-Infected-Susceptible (SIS) model fit for age-dependent seroprevalence of *Toxoplasma gondii* in humans, Europe, 2000–2021

The average *T. gondii* seroprevalence per region and by age derived from the model is presented in Figure 1 and Table 2. The United Kingdom (UK) was initially a country included in the western region. However, the analysis per region and age showed a deviant seroprevalence for the UK compared with the other countries included in our categorisation of western Europe. Therefore, the UK was analysed separately. The highest prevalence estimates were seen in eastern (50%), western (48%), southeastern (45%) and southwestern (38%) Europe. The seroprevalence was markedly lower in northern Europe (18%) and the UK (18%). Thus, while the seroprevalence increased with age from 13% to 16% in those aged ≤ 25 years to above 50% in those aged > 50 years in most regions, it increased from 4% to 26–27% in northern Europe and the UK.

In total, 69 studies with data from 25 countries were included in the model. The line indicates the fitted seroprevalence by age, with the orange area being the 95% confidence interval. The grey dots represent seroprevalence data points at the best estimate of age in the data for the studied populations. The size of the dots reflects the number of persons tested. The dots are shifted horizontally along the grey lines extending from the minimum to the maximum possible age, to the best fitting age (coloured dots).

**Table 2:** Modelled average seroprevalence estimates of *Toxoplasma gondii* in humans, by age group and age at infection, Europe, 2000–2021

Region^a^	Seroprevalence (%), by age group			Age at infection (years)			
	0–25 years	26–50 years	> 50 years	Mean	10% quantile	Median	90% quantile
Eastern	16	43	68	63.96	6.74	44.34	147.28
Northern	4	14	27	249.95	26.33	173.25	575.53
Southeastern	13	38	62	77.19	8.13	53.51	177.75
Southwestern	10	30	52	101.66	10.71	70.46	234.08
UK	4	13	26	259.07	27.30	179.58	596.54
Western	15	41	66	68.46	7.21	47.45	157.63
Overall	NA			136.72	14.40	94.76	314.80

NA: not applicable; UK: United Kingdom.

^a^ Eastern: Belarus, Czechia, Estonia, Hungary, Latvia, Lithuania, Poland, Romania, Slovakia, Ukraine; Northern: Denmark, Finland, Iceland, Norway, Sweden; Western: Austria, France, Germany, Ireland, Liechtenstein, Luxembourg, the Netherlands, Switzerland; Southeastern: Albania, Bosnia and Herzegovina, Bulgaria, Croatia, Cyprus, Greece, Kosovo, Moldova, North Macedonia, Serbia, Slovenia; Southwestern: Andorra, Italy, Malta, Portugal, San Marino, Spain.

A total of 69 studies were included in the analysis (two studies with results for more than one region): eastern (26 studies, 7 countries), northern (4 studies, 4 countries), southeastern (9 studies, 5 countries), southwestern (18 studies, 3 countries), western (11 studies, 5 countries), UK (3 studies).

**Figure 2:**
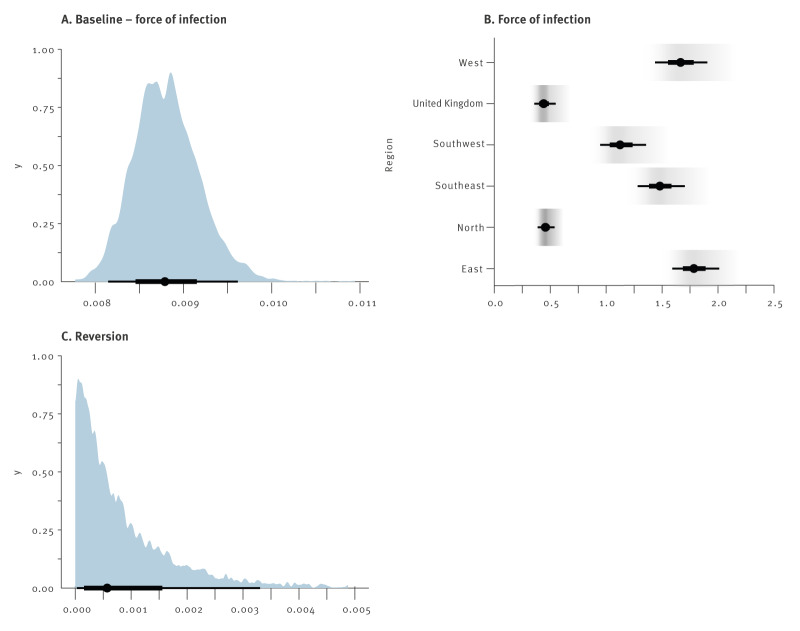
Bayesian hierarchical model outcomes (i.e. posterior probabilities) for the force of infection (λ) and reversion rate (γ) to seronegative status of *Toxoplasma gondii* in humans, Europe, 2000–2021

In addition to regional seroprevalence estimates, the model also provides estimates for the force of infection and the rate of reversion to seronegative status (Figure 2). For the force of infection, the inverse of the posterior coefficient represents the average waiting time in years until the event. For *T. gondii* infection, this results in an average waiting time of 1/0.009 ca 137 years (Figure 2), with a median waiting time of 95 years (Table 2). The lowest force of infection in Europe was observed in the UK (exp(λ_region_) = 0.445) and the northern region (exp(λ_region_) = 0.459), followed by the southwestern (exp(λ_region_) = 1.13) and the southeastern region (exp(λ_region_) = 1.48). Highest forces of infection were seen for the western (exp(λ_region_) = 1.67) and the eastern region (exp(λ_region_) = 1.79).

Values for the force of infection in the regions are all exponentiated, which means that a value of 1 indicates the absence of an effect on the baseline probability. Grey area represents the uncertainty distribution, the thin and thick black lines indicate the 95% and 50% Bayesian credible intervals, respectively, with dots indicating the mean of exponentiated forces of infection per region. In total, 69 studies with data from 25 countries were included in the model.

To reconstruct the total force of infection per year for each region, the baseline force of infection was multiplied by the region-specific exponentiated contributions. For example, in the eastern region, the average time until infection was estimated to be ca 64 years (1/(0.009 × 1.79)), with 50% of the population becoming infected by age 44 years (the 50% quantile of the exponential distribution with parameter 0.009 × 1.79). In contrast, the average time until infection in the northern region and the UK exceeded 250 years, and the time 50% of the population becoming infected exceeding 170 years (Table 2). Despite this long average, 10% of the population would be infected at the age of 26 years, reflecting the skewed distribution of the infection age distribution.

The reversion rate was estimated at γ = 9.0 × 10^−4^ (3.0 × 10^−4^, 3.5 × 10^−3^), which sets an average waiting time of > 1,000 years. However, at the upper end of the credible interval this average waiting time was 275 years, with 10% of the population reverted at 29 years and 20% at 60 years. Hence, considerable reversion is realistic within the credible interval.

## Discussion

The aim of this study was to model the age-dependent prevalence of *T. gondii* in the human population in Europe. To achieve this, a systematic review of seroprevalence studies in humans published in 2000–2021 was conducted. Data were extracted from 69 papers selected from 1,822 publications identified by the search strategy and, where possible, analysed using a Bayesian hierarchical model. Compared with conventional analytical techniques, Bayesian methods may perform better in meta-analyses, particularly by dealing better with uncertainty [84]. This is especially important when analysing population-based studies, where heterogeneity may arise from factors such as study design, geographic region, serological test used or missing data.

Human *T. gondii* seroprevalence data were obtained for 25 of the 41 countries included in the search. A similar geographic coverage of European *T. gondii* seroprevalence studies was achieved in a previous systematic review [85], indicating geographic gaps in available data. Meta-analysis was performed on a regional scale, by aggregating countries into five geographic areas. Albeit at the cost of loss of detail, grouping the data allowed for a broader geographic coverage, making it easier to identify regional differences and establish regional correlations. By using partial pooling of prevalence distributions from regions with a larger amount of data, the Bayesian hierarchical model allowed to provide seroprevalence estimates for regions lacking data, though with a larger uncertainty. Furthermore, age information given in the studies was notoriously incomplete, with differing age ranges used to categorise data, missing age data or use of categories that only gave an indication of age (e.g. children or pregnant women). The Bayesian hierarchical model helped to address these gaps by incorporating uncertainty on this variable, which made it possible to derive posterior predictive distributions for the age-dependent seroprevalence in each region in Europe. Modelling the prevalence of *T. gondii* by age provides a better understanding of infection dynamics, from birth to any given age, and holds the potential for developing age-specific prevention strategies. Thus far, most systematic review and meta-analysis studies have focused primarily on identifying sources of infection in outbreaks [86] and sporadic toxoplasmosis [3] or assessing prevalence in specific risk groups [87,88]. To our knowledge, one other study has reviewed seroprevalence data for the European general population [85], but age was not considered in the subgroup analysis in that study.

When estimating the age-dependent seroprevalence of *T. gondii*, we used the SIS model which includes the possibility that individuals can return to the susceptible state (some time) after infection. This approach was used before to model the age-dependent prevalence of *T. gondii* in animals [13]. In that study, the plateau in seroprevalence observed in animals at higher age was better explained by the SIS model, than the SI (Susceptible-Infected) model (where 100% of susceptible individuals would become infected if living long enough). Although hardly reported in literature, evidence of seroreversion in humans was, for example, reported in a cohort of blood donors followed over 4 years [23]. Nevertheless, the reversion rate estimated in the present study was negligible, supporting the hypothesis of lifelong persistence of anti-*T. gondii* antibodies in humans [89].

Results from this study revealed considerable differences in seroprevalence between geographic regions across Europe. The estimates for *T. gondii* seroprevalence were highest in eastern, western, and southeastern Europe, with a mean seroprevalence of 45–50%, followed by the southwestern region (38%), and were lowest in the UK and northern Europe where the model predicted a mean infection rate of 18%. Based on our results, seroprevalence in the age group 25–50 years varied between 13% and 43% in Europe. Worldwide, highest seroprevalence in pregnant women has been observed in South America (53–56%), mostly based on Brazil, and Africa (47–49%); the seroprevalence in Europe in this group was 25–31%, whereas the seroprevalence in North America was 20–28% [87,90,91]. Importantly, measured seroprevalences between countries within a continent can vary largely, as they can even within countries [92]. In line with the assumption of lifelong infection, the seroprevalence increased between the three age groups, with as few as 4% of individuals seropositive in the youngest age group in the UK and northern Europe and a several-fold increase up to 68% by the age of > 50 years in eastern Europe. Measuring the force of infection, i.e. the rate at which individuals acquire infection, is a key to understanding the epidemiology of infectious diseases and estimating disease burden. Estimates for this parameter clearly showed that individuals in eastern and western Europe became infected at a younger age, compared with the other regions. Translated into time until infection, this means that on average half of the population in eastern and western Europe was expected to be infected by the age of 47 years, while in northern Europe and the UK this was expected at an unreachable age of 173 and 179 years, respectively. Noteworthily, the strong force of infection estimated for western Europe is in agreement with the results from a recent large-scale *T. gondii* serosurvey in female children and adolescents in Germany, showing that with each year of life the chance of becoming seropositive increased by 1.2 [93].

Comparing *T. gondii* prevalence and force of infection in humans and animals in the same geographic area may provide One Health insights into potential sources. Interestingly, results obtained for humans in the present study parallel the force of infection trends among geographic regions disclosed in the previous review of the animal prevalence in Europe [13], except for western Europe, where the force of infection ranked second highest in humans but was lowest in animals. Although results from the two studies do not provide direct evidence of specific sources of human infection, a high prevalence in herbivorous animals that have outdoor access or are raised as free-range (e.g. sheep, wild ruminants) suggests that environmental contamination of *T. gondii* is a contributing factor [94,95]. While there is a possible association between high prevalence in animals with outdoor access and increased risk of human exposure from the environment, this does not necessarily translate into a higher seroprevalence in humans, as direct environmental exposure is not the only possible route of infection to humans.

To understand seroprevalence differences between countries or regions, more data are needed on the cultural differences in behaviours that can influence the risk of *T. gondii* infection. Seroprevalence in humans is likely influenced by consumption habits and consumed products, including imported products from other countries. For example, the frequency, amount, preparation and types of meat or meat products consumed, and especially local preferences for specific products made of raw or undercooked meat are important. Previously, two quantitative microbiological risk assessment (QMRA) models suggested that *filet américain*, a typical Dutch raw beef spread, was the most important source of *T. gondii* infection in the Netherlands [96,97]. Similarly, *Hackepeter* or *Mett*, a German dish made of raw minced pork meat, is more popular and consumed more frequently in eastern Germany compared with western Germany. This dietary preference was linked to a higher *T. gondii* seroprevalence in the human adult population in eastern Germany [81]. Moreover, exposure to oocysts can vary locally due to differences in soil exposure, frequency of consumption of raw vegetables, fruits and shellfish, or drinking water treatment [89].

Yet, studies assessing the relative contribution of the various sources of infection in Europe are scarce. Underlining the varying importance of animals raised for human consumption in the transmission of *T. gondii*, a previous multicentre case-control study attributed 30–63% of infections to the consumption of undercooked or cured meat products and 6–17% to soil contact [98]. Also, several studies identifying significant foodborne risk factors have found a link between the consumption of livestock-derived foods, such as raw or undercooked meat [17,21,34,37,40,50,77,99], types of processed meat [43,77], unpasteurised milk and raw milk cheese [17,34], and *T. gondii* infection. With regards to oocyst-driven infections, eating raw or unwashed vegetables or fruits [17,43], contact with cats [17,37,40,50,55,77,81] and contact with soil [21,43,66,74,77] were reported as risk factors. However, for a risk factor to be an important source of infection at a population level, exposure to the factor also needs to be common. Exposure behaviour can vary between and within populations, and these data are often lacking. The recent implementation of a harmonised European survey tailored to capture variation in the frequency and amount of meat and vegetable products of known risk consumed, as well as consumers’ behaviour associated with an increased risk of infection (e.g. preference for raw or undercooked meat, washing of vegetables) will bring new data for *T. gondii* food-borne risk assessment (https://onehealthejp.eu/projects/foodborne-zoonoses/jrp-toxosources).

In this study, only region and age were included in the model. Any differences related to sex will therefore be missed. Nevertheless, in most studies reporting results on sex, differences between sexes were not observed [15,17,70,100], except for one study where males were more often seropositive [81]. Other study limitations were mainly related to the absence of seroprevalence data for some European countries, the small scale of some studies and incomplete or inconsistent data reporting. Also, policies towards screening for *T. gondii* infections differ between countries. Although the Bayesian model could address uncertainty in both prevalence and age data, the lack of diagnostic performance characteristics of in-house serological tests and commercial kits used in the selected studies made it impossible to estimate ‘true prevalence’ [101]. To fill this gap, data on test sensitivity and specificity could potentially be sourced from validation studies by the manufacturer or diagnostic accuracy studies. However, previous attempts to retrieve this information from the literature have shown inconsistent results due to a variety of factors, including the characteristics of the sample population (e.g. immune status, time since infection, potentially cross-reacting pathogens or rheumatic factors), study design, quality of the reference standard used, or different cutoffs employed [102,103]. Thus, even when data on test characteristics are available, it is important to keep in mind that such data may not be constant over populations and that the reproducibility of serological test results is not warranted [102,104,105].

For future work to overcome the hurdles associated with data heterogeneity in serological studies, we emphasise the importance of the primary data source when publishing original research. A main challenge is assuring that raw data are reported in a way that allows their accessibility and their further reuse in reviews and meta-analyses [106]. A solution to this challenge is the development and adoption of standardised templates that enable harmonised reporting of data. Ideally, results from serological surveys should be provided on an individual basis, when possible, and include demographic information on age and sex, together with test performance characteristics, cutoffs, as well as titres or OD values. Additional details are desirable, e.g. the ELISA plate identification or analysis date, which allows for plate-to-plate correction when using binary mixture models [107]. A spreadsheet for epidemiological data reporting that could function as such a template was previously proposed [13]. These data templates could be used, for example, in the online supplementary files of research papers or made available in public repositories.

## Conclusion

Knowing the prevalence of infection is key information in assessing disease burden and estimating costs of illness and prevention efforts in a population. The results of this review revealed a considerably higher seroprevalence in eastern and western Europe, compared with northern Europe and the UK, and that *T. gondii* infection is occurring at an earlier age in these regions. The commonly held belief that the infection is lifelong is corroborated by our results, as the current best estimate of the reversion rate equates to infection durations that far exceed a human lifespan. However, the uncertainty in the estimate is large, and reversion rates that correspond to infection durations of decades are within the 95% credible interval. Prevalence distributions derived by the model contribute to a better understanding of disease burden caused by *T. gondii* and provide a baseline for future epidemiological research in the different European regions.

## Data Availability

Supplementary data for the reported results, including publicly archived datasets analysed or generated during the study, can be found at GitHub, an online data repository, reachable through the following URL: https://github.com/rivm-syso.

## Supplementary Data

137000 Supplementary Material
