# Supplementary material for: Systematic review and modelling of Toxoplasma gondii seroprevalence in humans, Europe, 2000 to 2021
Source: Euro Surveill. 2025 Aug 28;30(34):2500069. doi: 10.2807/1560-7917.ES.2025.30.34.2500069 (PMC12397722; doi:10.2807/1560-7917.ES.2025.30.34.2500069)
Supplement: Supplementary Material [file 25-00069_FRIESEMA_Supplement.pdf]

Supplementary material to the article: "Systematic review and modelling of human prevalence of Toxoplasma gondii in Europe"

This supplementary material is hosted by *Eurosurveillance* as supporting information alongside the article "Systematic review and modelling of human prevalence of Toxoplasma gondii in Europe", on behalf of the authors, who remain responsible for the accuracy and appropriateness of the content. The same standards for ethics, copyright, attributions and permissions as for the article apply. Supplements are not edited by *Eurosurveillance* and the journal is not responsible for the maintenance of any links or email addresses provided therein.

Table S1: Search string used to identify publications on human seroprevalence and risk factors of infections with *T. gondii* in Europe, 2000-2021.

No.	Query
#29	#28 AND ('article'/it OR 'article in press'/it OR 'review'/it)
#28	(#25 OR #26) AND [2000-2021]/py
#27	#25 OR #26
#26	#15 NOT #24
#25	#15 AND #16
#24	#17 OR #18 OR #19 OR #20 OR #21 OR #22 OR #23
#23	'brazil*':ti OR 'russia*':ti OR 'australia*':ti OR 'japan*':ti OR china:ti OR chinese:ti OR 'colombi*':ti OR 'iran*':ti OR galapagos:ti OR 'korea*':ti OR 'saudi arabia*':ti OR 'mexic*':ti OR 'india*':ti OR 'indonesia*':ti OR peru:ti OR iraq:ti OR mongolia:ti OR thailand:ti OR 'pakistan*':ti OR 'angol*':ti OR 'south africa*':ti OR usa:ti OR 'united states':ti OR 'algeria*':ti OR qatar:ti OR kenya:ti OR 'ethiopia*':ti
#22	'arctic and antarctic'/exp OR arctic:ti OR antarctic:ti
#21	'oceanic regions'/exp
#20	'western hemisphere'/exp
#19	'australia and new zealand'/exp OR 'australia*':ti OR 'new zealand':ti
#18	'asia'/exp OR asia:ti
#17	'africa'/exp OR 'africa*':ti
#16	'europe'/exp OR 'europ*':ti,ab OR france:ti,ab OR french:ti,ab OR 'german*':ti,ab OR netherlands:ti,ab OR dutch:ti,ab OR 'belgi*':ti,ab OR spain:ti,ab OR spanish:ti,ab OR italy:ti,ab OR italian:ti,ab OR switzerland:ti,ab OR swiss:ti,ab OR 'austria*':ti,ab OR 'bulgaria*':ti,ab OR 'hungar*':ti,ab OR ireland:ti OR irish:ti,ab OR 'united kingdom':ti,ab OR 'croatia*':ti,ab OR poland:ti,ab OR polish:ti,ab OR 'czech*':ti,ab OR portugal:ti,ab OR 'portugues*':ti,ab OR liechtenstein:ti,ab OR luxembourg:ti,ab OR denmark:ti,ab OR danish:ti,ab OR finland:ti,ab OR finnish:ti,ab OR iceland:ti,ab OR norway:ti,ab OR sweden:ti,ab OR swedish:ti,ab OR estonia:ti,ab OR 'latvia*':ti,ab OR 'lithuania*':ti,ab OR 'moldova*':ti,ab OR 'romania*':ti,ab OR 'slovakia*':ti,ab OR ukraine:ti,ab OR belarus:ti,ab OR andorra:ti,ab OR malta:ti,ab OR monaco:ti,ab OR 'san marino':ti,ab OR 'albania*':ti,ab OR 'bosnia and herzegovina':ti,ab OR bosnian:ti,ab OR cyprus:ti,ab OR greece:ti,ab OR 'greek*':ti,ab OR 'kosovo*':ti,ab OR 'republic of macedonia':ti,ab OR montenegro:ti,ab OR 'serbia*':ti,ab OR 'slovenia*':ti,ab OR corsica:ti,ab OR madeira:ti,ab OR azores:ti,ab OR gibraltar:ti,ab OR sardinia:ti,ab OR sicily:ti,ab OR 'canary island*':ti,ab OR 'channel island*':ti,ab OR guernsey:ti,ab OR jersey:ti,ab OR 'isle of man':ti,ab OR 'scandinavia*':ti,ab OR 'faroe islands':ti,ab OR england:ti,ab OR scotland:ti,ab OR wales:ti,ab OR 'great britain':ti,ab OR mallorca:ti,ab OR menorca:ti,ab OR ibiza:ti,ab OR formentera:ti,ab OR tenerife:ti,ab OR fuerteventura:ti,ab OR 'gran canaria':ti,ab OR lanzarote:ti,ab OR 'la palma':ti,ab OR 'la gomera':ti,ab OR 'el hierro':ti,ab OR 'la graciosa':ti,ab OR aland:ti,ab
#15	#9 OR #10 OR #12 OR #14
#14	#8 AND #13
#13	'population'/exp
#12	#8 NOT #11
#11	#8 AND [animals]/lim

#10	#8 AND [clinical study]/lim
#9	#8 AND [humans]/lim
#8	#4 AND (#5 OR #6 OR #7)
#7	'risk'/exp AND 'factor*':ti,ab
#6	'risk factor'/exp/mj OR 'risk factor*':ti
#5	seroprevalence'/exp OR 'seropreva*':ti,ab OR 'sero-preva*':ti,ab OR 'prevalence'/exp/mj OR 'prevalen*':ti OR 'virus detection'/exp OR 'seroconversion'/exp OR 'serodiagnosis'/exp OR 'seroepidemiology'/exp OR 'sero*':ti OR 'epidemiological data'/exp/mj OR 'epidemiology'/exp/mj
#4	#1 OR #2 OR #3
#3	'toxoplasma gondii':ti,ab
#2	'toxoplasma'/exp OR 'toxoplasma*':ti
#1	'toxoplasmosis'/exp OR 'toxoplasmo*':ti

Table S2: Categorization of European countries into five European regions

**Regions**	**Countries**
Western	Austria, France, Germany, Ireland, Liechtenstein, Luxembourg, the Netherlands, Switzerland, the United Kingdom
Northern	Denmark, Finland, Iceland, Norway, Sweden
Eastern	Belarus, Czechia, Estonia, Hungary, Latvia, Lithuania, Poland, Romania, Slovakia, Ukraine
Southeastern	Albania, Bosnia and Herzegovina, Bulgaria, Croatia, Cyprus, Greece, Kosovo, Moldova, North Macedonia, Serbia, Slovenia
Southwestern	Andorra, Italy, Malta, Portugal, San Marino, Spain

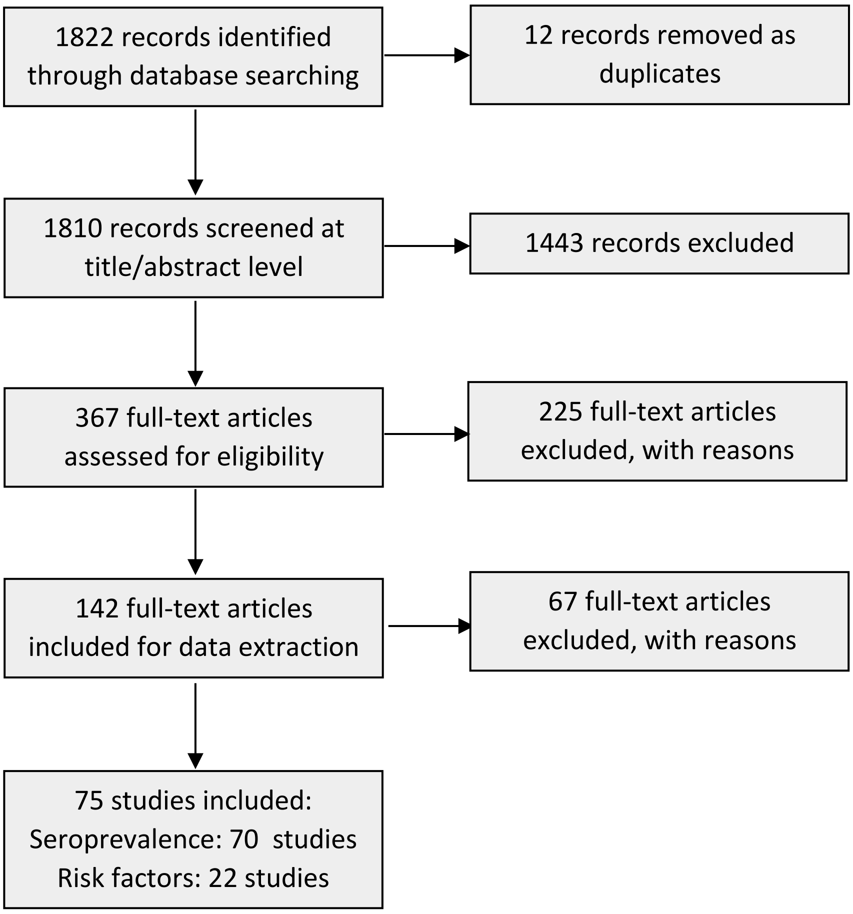

Figure S1: PRISMA flow diagram. The flow diagram represents graphically the screening steps and data extraction step with numbers of publications excluded and number of publications proceeding to the following step, leading to the final number of included articles.

References of 22 identified articles on risk factors

1. Asencio MA, Herraez O, Tenias JM, Garduão E, Huertas M, Carranza R, et al. Seroprevalence survey of zoonoses in extremadura, southwestern Spain, 2002–2003. Japanese Journal of Infectious Diseases. 2015;68(2):106-12.
2. Birgisdóttir A, Asbjörnsdottir H, Cook E, Gislason D, Jansson C, Olafsson I, et al. Seroprevalence of Toxoplasma gondii in Sweden, Estonia and Iceland. Scandinavian Journal of Infectious Diseases. 2006;38(8):625-31.
3. Bobić B, Nikolić A, Klun I, Vujanić M, Djurković-Djaković O. Undercooked meat consumption remains the major risk factor for Toxoplasma infection in Serbia. Parassitologia. 2007;49(4):227-30.
4. Flatt A, Shetty N. Seroprevalence and risk factors for toxoplasmosis among antenatal women in London: a re-examination of risk in an ethnically diverse population. European journal of public health. 2013;23(4):648-52.
5. Fromont EG, Riche B, Rabilloud M. Toxoplasma seroprevalence in a rural population in France: Detection of a household effect. BMC Infectious Diseases. 2009;9.
6. Hofhuis A, Van Pelt W, Van Duynhoven YTHP, Nijhuis CDM, Mollema L, Van Der Klis FRM, et al. Decreased prevalence and age-specific risk factors for Toxoplasma gondii IgG antibodies in The Netherlands between 1995/1996 and 2006/2007. Epidemiology and Infection. 2010:1-9.
7. Jerant PV, Milosević V, Hrnjaković Cvjetković I, Patić A, Stefan Mikić S, Ristić M. Toxoplasma gondii infection in pregnant women. [Infekcije toksoplazmom gondii kod gravidnih zena.] Medicinski pregled. 2013;66(11-12):459-63.

8. Juncker-Voss M, Prosl H, Lussy H, Enzenberg U, Auer H, Lassnig H, et al. Screening for antibodies against zoonoses among employees of the Zoological Garden of Vienna, Schönbrunn, Austria. [Untersuchungen auf antikörper gegen Zoonoseerreger bei Angestellten des Wiener Tiergartens Schönbrunn.] Berliner und Munchener Tierarztliche Wochenschrift. 2004;117(9-10):404-9.
9. Kolbekova P, Kourbatova E, Novotna M, Kodym P, Flegr J. New and old risk-factors for Toxoplasma gondii infection: Prospective cross-sectional study among military personnel in the Czech Republic. Clinical Microbiology and Infection. 2007;13(10):1012-7.
10. Lobo ML, Patrocinio G, Sevivas T, De Sousa B, Matos O. Portugal and Angola: Similarities and differences in Toxoplasma gondii seroprevalence and risk factors in pregnant women. Epidemiology and Infection. 2017;145(1):30-40.
11. Lopes AP, Dubey JP, Moutinho O, Gargate MJ, Vilares A, Rodrigues M, et al. Seroepidemiology of Toxoplasma gondii infection in women from the North of Portugal in their childbearing years. Epidemiology and Infection. 2012;140(5):872-7.
12. Nash JQ, Chissel S, Jones J, Warburton F, Verlander NQ. Risk factors for toxoplasmosis in pregnant women in Kent, United Kingdom. Epidemiology and Infection. 2005;133(3):475-83.
13. Olariu TR, Ursoniu S, Hotea I, Dumitrascu V, Anastasiu D, Lupu MA. Seroprevalence and Risk Factors of Toxoplasma gondii Infection in Pregnant Women from Western Romania. Vector-Borne and Zoonotic Diseases. 2020;20(10):763-7.
14. Radon K, Windstetter D, Eckart J, Dressel H, Leitritz L, Reichert J, et al. Farming exposure in childhood, exposure to markers of infections and the development of atopy in rural subjects. Clinical and Experimental Allergy. 2004;34(8):1178-83.
15. Rodrigues FT, Sousa AP, Escoval MA, Condeço J, Cardoso L, Lopes AP. Seroepidemiology of Toxoplasma gondii in blood donors in Portugal. Transfusion and Apheresis Science. 2020;59(4).
16. Said B, Halsby KD, O'Connor CM, Francis J, Hewitt K, Verlander NQ, et al. Risk factors for acute toxoplasmosis in England and Wales. Epidemiology and Infection. 2017;145(1):23-9.
17. Salamon D, Bulanda M. Toxoplasma gondii and women of reproductive age: an analysis of data from the Chair of Microbiology, Jagiellonian University Medical College in Cracow. Annals of parasitology. 2014;60(4):291-6.
18. Siponen AM, Kinnunen PM, Koort J, Kallio-Kokko H, Vapalahti O, Virtala AM, et al. Toxoplasma gondii seroprevalence in veterinarians in Finland: Older age, living in the countryside, tasting beef during cooking and not doing small animal practice associated with seropositivity. Zoonoses and Public Health. 2019;66(2):207-15.
19. Sroka J, Zwoliński J, Dutkiewicz J. The prevalence of anti-Toxoplasma gondii antibodies among abattoir workers in Lublin. [Czekstość wystepowania przeciwciał anty-Toxoplasma gondii wśród pracowników Zakładów Miesnych w Lublinie.] Wiadomości parazytologiczne. 2003;49(1):47-55.
20. Studenićová C, Ondriska F, Holková R. Seroprevalence of Toxoplasma gondii among pregnant women in Slovakia (Séroprevalencia Toxoplasma gondii u gravidných žien na Slovensku). Epidemiologie, Mikrobiologie, Imunologie. 2008;57(1):8-13.
21. Thaller R, Tammaro F, Pentimalli H. Risk factors for toxoplasmosis in pregnant women in central Italy (Fattori di rischio per la toxoplasmosi in gravidanza in una popolazione del centro Italia). Infezioni in Medicina. 2011;19(4):241-7.
22. Wilking H, Thamm M, Stark K, Aebischer T, Seeber F. Prevalence, incidence estimations, and risk factors of Toxoplasma gondii infection in Germany: a representative, cross-sectional, serological study. Scientific reports. 2016;6:22551.
